# The Potential Mechanism Behind Native and Therapeutic Collaterals in Moyamoya

**DOI:** 10.3389/fneur.2022.861184

**Published:** 2022-04-26

**Authors:** Xiang-Yang Bao, Yan-Na Fan, Qian-Nan Wang, Xiao-Peng Wang, Ri-Miao Yang, Zheng-Xing Zou, Qian Zhang, De-Sheng Li, Lian Duan, Xin-Guang Yu

**Affiliations:** ^1^Department of Neurosurgery, Chinese People's Liberation Army of China (PLA) General Hospital, Beijing, China; ^2^Chinese PLA Medical School, Beijing, China; ^3^Department of Radiation Oncology, The Fifth Medical Center of Chinese PLA General Hospital (Former 307th Hospital of the PLA), Beijing, China

**Keywords:** moyamoya disease, *RNF213* p.R4810K, clinical phenotype, collaterals, mutation

## Abstract

**Background and Purpose:**

To explore the genetic basis and molecular mechanism of native arteriogenesis and therapeutic synangiosis in moyamoya disease (MMD).

**Methods:**

An angiography-based study using patients from a prospective trial of encephaloduroarteriosynangiosis (EDAS) surgery was performed. The spontaneous collaterals grades were evaluated according to the system described by a new grading system. Blood samples were collected from all the recruited patients before EDAS and during the second hospitalization 3 months post-EDAS. We performed Boolean analysis using a combination of specific cell surface markers of CD34^bri^CD133^+^CD45^dim^KDR^+^. Genotyping of p.R4810K was also performed. The correlation of age, sex, initial symptoms at diagnosis, collateral grade, Suzuki stages, the RNF213 genotype, time to peak (TTP), and endothelial progenitor cell (EPC) count with good collateral circulation was evaluated.

**Results:**

Eighty-five patients with MMD were included in this study. The mutation rate of RNF213 p.R4810K in our study was 25.9% (22/85). The heterozygous mutations were occurred significantly more frequently in the cases that were presented with infarction, worse neurological status, severe posterior cerebral artery (PCA) stenosis, and longer TTP delay. Further, the heterozygous mutations occurred significantly more frequently in the poor collateral stage group. Lower grades were significantly correlated with severe ischemia symptoms, worse neurological status, and a longer TTP delay. The post-operative angiographic findings showed that a good Matsushima grade was correlated with heterozygous mutations, a lower collateral stage, and a longer TTP delay. The CD34^bri^CD133^+^CD45^dim^KDR^+^ cell count in patients 3 months post-EDAS was significantly higher as compared to the count before EDAS in the good Matsushima grade group. However, this change was not observed in the poor Matsushima grade group.

**Conclusions:**

These data imply that mutations of RNF213 p.R4810K affect the establishment of spontaneous collateral circulation, and EPCs are involved in the process of formation of new EDAS collaterals.

## Introduction

Moyamoya disease (MMD) is a rare, chronic, and progressive cerebrovascular disorder that is characterized by stenosis and occlusion of the distal carotid, proximal, middle, and anterior cerebral arteries and is accompanied by the development of a fine collateral vascular network at the base of the brain (moyamoya vessels) (1, 2). Besides, patients with MMD can develop spontaneous collaterals (innate collateral vessels) in areas of ischemia. These alternative conduits vary from individual to individual, resulting in different clinical presentations, the severity of disease, and outcomes. Successful compensatory collateralization is considered a defensive measure against ischemic and hemorrhagic stroke in MMD (3, 4). These spontaneous collaterals are of such importance that, to some extent, they determine, or rather indicate, the fate of patients with MMD. Although many articles have reported relevant factors that affect the formation of these collateral vessels in MMD, the mechanisms involved in spontaneous vessels formation are not fully understood.

Indirect revascularization is an essential component of the surgical treatment of MMD. This procedure improves cerebral perfusion by attaching pedicled, vascularized grafts to the cortical surface, and facilitating the in-growth of angiogenesis. Compared to direct revascularization, indirect revascularization is a much simpler, less strenuous technique with lower risks of post-operative complications, and as demonstrated in many recent studies, may achieve long-term outcomes as satisfying as those for a direct bypass surgery (5–9). However, the mechanisms involved in synangiosis induced by indirect revascularization are also not well-understood. The uncertainty of post-surgical synangiosis is the main consideration that limits the use of this method. According to earlier reports, collateral vessel formation in encephaloduroarteriosynangiosis (EDAS), a commonly used indirect revascularization surgery, may be primarily driven by angiogenesis (10, 11). Angiogenesis is induced by hypoxia and involves the formation of new collateral vessels by sprouting or splitting from the pre-existing vascular structures (12, 13). Studies have shown that a subtype of immature circulating cells is involved in endothelial repair, neo-angiogenesis, and tissue homeostasis (14, 15). The surface markers used to identify such endothelial progenitor cells (EPCs) have not been consistent across studies, creating confusion (16). However, phenotypical and functional tests show that CD34^bri^CD133^+^KDR^+^ cells are a population of endothelial precursor cells (17). Based on previous studies (18, 19), here we used the markers CD34^bri^CD133^+^CD45^dim^KDR^+^ to define putative EPCs, although this definition is not consensual.

Genetics has contributed greatly to understanding the pathophysiology of MMD. A polymorphism, R4810K (p.Arg4810Lys), in the Ring Finger Protein 213 (RNF213) gene was identified as the strongest genetic susceptibility factor for MMD in East Asian populations, which could increase the risk of MMD by ≈300 times (20, 21). Moreover, patients with a homozygous RNF213 mutation are predisposed to significantly early-onset age, rapid disease progression, cerebral infarction at diagnosis, and poor prognosis (22–24). Thus, RNF213 is a major susceptibility gene for MMD, and it has been recognized as a key molecule to understand the pathophysiology of MMD. *In vitro* and *in vivo* experiments revealed that RNF213 is related to angiogenesis and vascular inflammation; however, the exact physiologic functions of RNF213 remain unknown (25).

Therefore, we designed an angiography-based study using patients from a prospective trial of EDAS surgery to study the locus (c.14429G>A) p.R4810K of the *RNF213* and EPCs in patients with MMD, in order to understand the genetic basis and molecular mechanism of these two types of collateral vessels in MMD (native arteriogenesis and therapeutic synangiosis).

## Methods

### Patient Selection

We prospectively recruited patients who were diagnosed with MMD at the Department of Neurosurgery in the Fifth Medical Center of Chinese PLA General Hospital, Beijing, China between 2018 and 2019. The diagnosis was based on the 2012 guidelines for MMD (26). Patients with moyamoya syndrome secondary to the identified etiologies were ruled out. As circulating EPC counts are affected by age according to previous reports (27, 28), we only collected adult patients who were aged 30–35 years to minimize this effect. Patients in whom MMD was associated with endothelial injuries, such as trauma, cancer, antiphospholipid antibodies, and surgery within the past 3 months, were excluded from the study, as well as individuals with pregnancy, renal disease, or hepatic disease. All patients enrolled in the study had received EDAS, which was performed by a single surgeon. In order to avoid the occurrence of post-operative stroke, we usually require patients to undergo vascular reconstruction surgery after 3 months of cerebral infarction or cerebral hemorrhage. The surgical procedure was as described in our previous reports (8, 9, 29). Bilateral surgery or more is generally required for patients with MMD. To eliminate the interference of surgery on the contralateral cerebral hemisphere, we only enrolled patients who were undergoing the first revascularization. The study was approved by the Research Ethics Board at the Fifth Medical Center of Chinese PLA General Hospital and all the subjects provided written informed consent to participate in the study. All the procedures performed in this study that involved human participants were in accordance with the Declaration of Helsinki (1964). This study was registered at ClinicalTrials.gov (NCT03613701).

### Clinical Data Collection

Information on patient sex, age at admission, family history of MMD, clinical history [hypertension, hyperlipidemia, and diabetes mellitus (DM)], initial symptomatic presentation at diagnosis, radiographic presentations at admission, hemodynamic status, neurological status, and perioperative complications was recorded. A symptomatic hemisphere was defined as a hemisphere with the most severe neurological symptom, and these symptoms were classified into (a) cerebral infarction; (b) intracranial hemorrhage/intraventricular hemorrhage; and (c) transient ischemic attack (TIA), seizures, and headache. Headache or seizures that could not be clearly localized to only one hemisphere was attributed to both hemispheres. Thus, both hemispheres from 11 patients who showed seizures or headaches were included in our study. Neurological status was evaluated with the modified Rankin Scale (mRS) on admission, which was classified as good (mRS score ≤ 2) or poor (mRS score ≥ 3).

The cerebral hemodynamic status was assessed by dynamic susceptibility contrast-magnetic resonance imaging (DSC-MRI) examination using a MAGNETOM Skyra 3T MRI scanner (Siemens, Germany) following previously described methods (30). The acquired DSC-MRI images were processed using a post-processing workstation (Syngo Via 20, Siemens) and analyzed with the MR Neuro-Perfusion software. We used the time to peak (TTP; the time at which contrast level reaches its maximum) and the mean relative cerebral blood volume (rCBV), a hemodynamic parameter calculated by MR perfusion, to evaluate the hemodynamic status of the patients.

### Angiography Finding

Digital subtraction angiography (DSA), such as injection of both internal carotid arteries and both vertebral arteries and assessment through the late venous phase to evaluate the collateral flow from all possible sources, was required for all recruited patients pre-operatively and on the second hospitalization 3 months post first EDAS. The angiographic collateral grade was evaluated according to the system described in our recent study (31). The grading score was obtained based on the collateral circulation and ranged from 1 to 12 as follows. (1) The anatomy extent of pial collateral blood from posterior cerebral artery (PCA) territory to the anterior cerebral artery (ACA) and middle cerebra artery (MCA) territory during the delayed venous phase. (2) The basal brain perforators and moyamoya vessels were defined using the Suzuki stage. The three collateral circulation status stages in MMD were defined as follows: scores of 1–4 were defined as a poor collateral status (stage I), scores of 5–8 were defined as a fair collateral status (stage II), and scores of 9–12 points were defined as a good collateral status (stage III). All angiographic images were reviewed by two experienced readers (Xiang-Yang Bao and Qian-Nan Wang) who were blinded to the angiography results and clinical details. Any differences in their observations were resolved by consensus.

The development of collateral circulation through EDAS was graded according to the system described by Matsushima et al. (32): Grade A, in which the area supplied by EDAS covered more than two-thirds of the MCA distribution; Grade B, in which one-third to two-thirds of the MCA distribution was covered; Grade C, in which only one-third of the MCA distribution was covered through EDAS, or no collateral circulation was observed. The patients were divided into a good post-operative collateral circulation group (grade A or grade B) and a poor post-operative collateral circulation group (grade C). Matsushima grades were also reviewed by two experienced readers (Xiang-Yang Bao and Qian-Nan Wang), and any differences in their observations were resolved by consensus.

### Blood Sampling and Flow Cytometric Analysis

Blood samples were collected from all the recruited patients before EDAS and during the second hospitalization 3 months post-EDAS. To evaluate the EPC count, blood samples were drawn from an antecubital vein. The venous blood was collected in 5 ml acid citrate dextrose tubes and processed within 3 h of collection. The blood samples were kept at 4°C throughout the procedure. The whole peripheral blood samples were analyzed by flow cytometry (33). A detailed description of the steps involved is described in our previous study (27). We performed Boolean analysis using the surface markers CD34^bri^CD133^+^CD45^dim^KDR^+^. All analyses were performed using FlowJo (Version 7.6 for MacIntosh, Tree Star Inc.). The CD34^bri^CD133^+^CD45^dim^KDR^+^ cell count was reported as a percentage of peripheral blood mononuclear cells (PBMCs).

### Quantification of EPC Count

The CD34+ cells were sorted from the peripheral blood, labeled with fluorochrome-conjugated monoclonal antibodies against CD34, CD45, CD133, and KDR [the human analog of vascular endothelial growth factor receptor-2 (VEGFR-2)] (34), and analyzed by flow cytometry. CD34^bri^CD133^+^CD45^dim^KDR^+^ cells were counted.

### DNA Extraction and Single Nucleotide Polymorphisms Genotyping

After receiving informed consent, 10 ml of peripheral vein blood was collected from the recruited patients with MMD, placed in EDTANa_4_ anticoagulant tubes, and stored in a freezer at −80°C until analysis. Genomic DNA was extracted from the blood samples using a Blood Genetic DNA Mini Kit (CWBIO, Beijing, China). The concentration of the DNA was measured using NanoDrop 2000 (Thermo Fisher Scientific, Waltham, MA, USA) and then diluted to a working concentration of 5 ng/μl for genotyping and validation. Genotyping of p.R4810K was performed following the protocol described in our previous study (35).

### Statistical Analysis

All the clinical characteristic data are presented as mean ± standard deviation (SD) for continuous variables and *n* (%) for categorical variables. The categorical variables were analyzed using the chi-square (χ^2^) test, and the continuous variables were compared using the independent Student's *t*-tests or analysis of variance (ANOVA). Mann-Whitney *U*-test was performed for variables that did not follow a normal distribution. The angiography outcomes were reviewed, and the correlation of age, sex, initial symptoms at diagnosis, collateral grade, Suzuki stages, the RNF213 genotype, TTP, and EPC count with good collateral circulation was evaluated using univariate analysis. The variables with significant association (*p* < 0.05) were selected and subjected to multiple linear regression analysis. The data were considered statistically significant when the value of *p* < 0.05. All statistical analyses were carried out with IBM SPSS statistical software version 22 for Windows (IBM Corp., Armonk, NY, USA).

### Data Availability Statement

With the permission of the corresponding authors and their institutions, combined with the relevant documents, all data used for analysis will be shared after ethics approval if requested by other investigators for reasonable purposes of replicating procedures and results.

## Results

### Patient Characteristics

A total of 137 newly diagnosed patients with MMD, aged 30–35 years at admission, who were treated at the Fifth Medical Center of Chinese PLA General Hospital between February 2018 and February 2019 were identified. Among the 137 patients, we excluded eight patients without pre-operative DSA, six asymptomatic patients who were not indicated for surgery, seven patients who refused surgical treatment, and 31 patients who refused follow-up DSA on the second hospitalization 3 months post first EDAS. Finally, 85 patients with MMD were included in this study.

Of the 85 patients, 49 (57.6%) were men and 5 (5.9%) had a family history of MMD. The mean age at admission was 33.01 ± 1.43 years. Thirty-eight patients were diagnosed with either hypertension, diabetes mellitus (DM), or hyperlipidemia. Fifteen patients had a history of smoking. TIA (30, 35.2%) and infarction (29, 34.1%) were the most common initial symptoms. Other symptoms included hemorrhage (15, 17.6%), headache (8, 9.4%), and seizures (3, 3.5%, Table 1).

**Table 1:** Demographic data of the 85 bilateral MMD patients.

**Variables**	**Value (%)**
Number of patients	85
Mean age ± SD (years)	33.01 ± 1.43
**Sex**	
Females	36 (42.4%)
Males	49 (57.6%)
History of risk factors	51
Hypertension	21
Diabetes mellitus	6
Hyperlipidemia	11
Smoking	15
Family history of MMD	5 (5.9%)
**Initial symptomatic presentation**	
TIA	30 (35.2%)
Infarction	29 (34.1%)
Hemorrhage	15 (17.6%)
Headache	8 (9.4%)
Seizures	3 (3.5%)
PCA involvement	24 (27.0%)
**Genotype of** ***RNF213*** **p.R4810K**	
G/A	22 (25.9%)
G/G	63 (74.1%)

*MMD, moyamoya disease; hps, hemispheres; pst, patients; TIA, transient ischemic attack; PCA, posterior cerebral artery*.

### The Genotype of *RNF213* p.R4810K

The mutation rate in cases carrying *RNF21*3 p.R4810K in our study was 25.9% (22/85). All the variant p.R4810K genotypes carried heterozygous mutations (G/A), and no homozygous mutations (A/A) were detected (Table 1). No significant difference in the sex, clinical history between the G/A genotype group and the G/G genotype group was noted. The heterozygous mutations were occurred significantly more frequently in the cases that were presented with PCA involvement (11/22, 50.0% vs. 13/63, 20.6%, *p* = 0.008). Similarly, the heterozygous mutations were significantly correlated with longer TTP delay (5.17 ± 2.61 s vs. 2.42 ± 1.53 s, *p* = 0.042). However, no significant difference in the CD34^bri^CD133^+^CD45^dim^KDR^+^ cell count between the G/A genotype group and the G/G genotype group was noted.

### Association Between MMD Collateral Grading and Factors

As per the definition provided in the methods section, a total of 96 symptomatic hemispheres were analyzed in the 85 patients. According to the new collateral grading system for MMD, there were 27, 36, and 33 hemispheres in the poor, fair, and good collateral stage groups, respectively (Table 2). Among 85 patients, 44% (11 of 25) of stage I hemispheres, 21.8% of stage II hemispheres (7 of 32), and 14.2% of stage III hemispheres (4 of 28) carried a heterozygous mutation (G/A). The heterozygous mutations were occurred significantly more frequently in the poor collateral stage group (*p* = 0.039). Among the 96 hemispheres, 55.5% (15 of 27) of stage I hemispheres, 25.0% of stage II hemispheres (9 of 36), and 15.1% of stage III hemispheres (5 of 33) were presented with infarction. Lower grades were significantly correlated with severe ischemia symptoms (*p* = 0.026, Table 2). Similarly, lower grades were significantly correlated with longer TTP delay and worse rCBV. The new collateral circulation scores in Suzuki grade were significantly different (*p* = 0.000), and high collateral scores were mostly distributed in Suzuki stage 3 or 4. However, no significant difference in the CD34^bri^CD133^+^CD45^dim^KDR^+^ cell count was noted among these three groups.

**Table 2:** Classification of intracranial collateral circulation and clinical presentation in 96 symptomatic hemispheres.

**Presentation**	**Grades**			**Total**	***P*-value**
	**Poor (stage I)**	**Fair (stage II)**	**Good (stage III)**		
Hemispheres	27 (28.1%)	36 (37.5%)	33 (34.4%)	96	
**Genotype of p.R4810K**					**0.039**
G/G	14 (56.0%)	25 (78.1%)	24 (85.7%)	63	
G/A	11 (44.0%)	7 (21.9%)	4 (14.3%)	22	
Clinical history	18 (66.7%)	18 (50.0%)	17 (51.5%)	53	0.366
**Symptomatic presentation**					**0.026**
TIA	6 (22.2%)	14 (38.9%)	10 (30.3%)	30	
Infarction	15 (55.6%)	9 (25.0%)	5 (15.2%)	29	
Hemorrhage	2 (7.4%)	5 (13.9%)	8 (24.2%)	15	
Headache, seizures	4 (14.8%)	8 (22.2%)	10 (30.3%)	22	
**Suzuki stages**					**0.000**
1	0	2 (5.6%)	3 (9.1%)	5	
2	0	4 (11.1%)	6 (18.2%)	10	
3	3 (11.1%)	5 (13.9%)	10 (30.3%)	18	
4	6 (22.2%)	6 (16.7%)	14 (42.4%)	26	
5	12 (44.4%)	11 (30.6%)	0	23	
6	6 (22.2%)	8 (22.2%)	0	14	
**Neurological status**					**0.092**
Poor	5 (20.0%)	2 (6.3%)	1 (3.6%)	8	
Good	20 (80.0%)	30 (93.8%)	27 (96.4%)	77	
DSC-MRI on admission	27	36	33	96	
TTP delay (seconds)	5.87 ± 2.69	3.81 ± 1.32	2.34 ± 1.06		0.001
rCBV ratio (%)	1.58 ± 0.61	1.63 ± 0.55	1.88 ± 0.65		0.028
Perioperative stroke (hps)	3 (3.7%)	1 (2.8%)	0	3	
EPCs before EDAS	0.076 ± 0.058	0.069 ± 0.053	0.065 ± 0.049		0.823
**Matsushima grade**					**0**
Grade A or grade B	21 (84.0%)	18 (56.3%)	7 (25.0%)	46	
Grade C	4 (16.0%)	14 (43.8%)	21 (75.0%)	39	

*hps, hemispheres; pst, patients; TIA, transient ischemic attack; IPH, intraparenchymal hemorrhage; IVH, intraventricular hemorrhage; mRS, Modified Rankin Scale; DSC-MRI, dynamic susceptibility contrast-magnetic resonance imaging; TTP, time to peak; rCBF, relative cerebral flow; rCBV, relative cerebral blood volume*.

### Factors Associated With Good Post-operative Collateral Circulation

**Figure 1:**
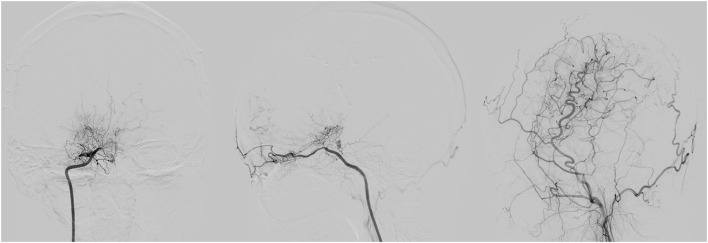
A 31 years old female with G/A genotype showed seriously ischemic situation with a poor collateral status (stage I) pre-operatively and surgical collateral circulation showed grade A 3 months after encephaloduroarteriosynangiosis (EDAS) procedure. The three illustrations of digital subtraction angiography (DSA) from left to right are the front view of right internal carotid artery (ICA), the vertical view of right ICA, the vertical view of right external carotid artery (ECA) 3 months after EDAS.

**Figure 2:**
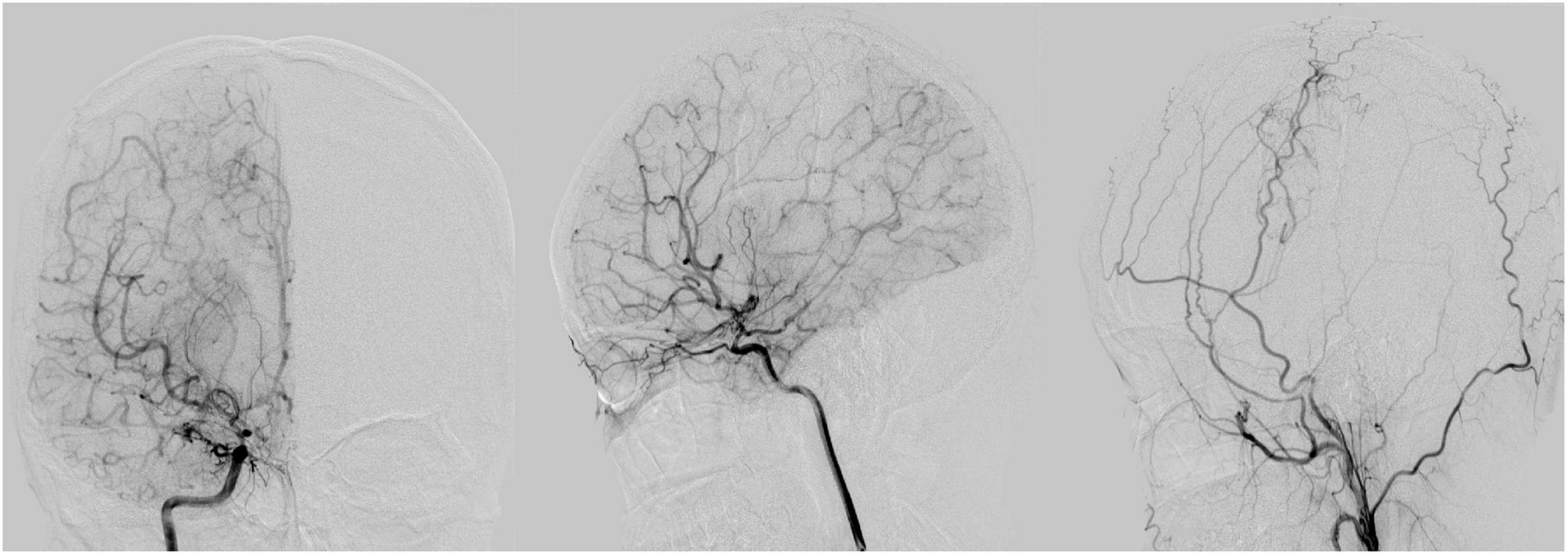
A 34 years old male as G/G genotype showed moderate ischemic condition with a good collateral status (stage III) pre-operatively and surgical collateral circulation showed grade C 3 months after encephaloduroarteriosynangiosis (EDAS) procedure. The three illustrations of digital subtraction angiography (DSA) from left to right is the front view of right internal carotid artery (ICA), the vertical view of right ICA, the vertical view of right external carotid artery (ECA) 3 months after EDAS.

Encephaloduroarteriosynangiosis was performed in all 85 patients, most of them were symptomatic hemispheres. The hemisphere with a longer TTP delay was selected for those patients who were presented with headaches and seizures. Critical complications, such as infarction, occurred in four hemispheres (three of stage I hemispheres and one of stage II hemispheres) during the perioperative period. The post-operative angiographic findings showed that a good Matsushima grade was correlated with a lower pre-operative collateral stage. Figures 1, 2 show two representative patients from poor and good groups. PCA involvement was occurred significantly more frequently in a good Matsushima grade group (*p* = 0.015, Table 3). Good Matsushima grade was correlated with longer TTP delay (*p* = 0.043, Table 3). Interestingly the CD34^bri^CD133^+^CD45^dim^KDR^+^ cell count in patients 3 months post-EDAS was significantly higher as compared to the count before EDAS in the good Matsushima grade group (0.126 ± 0.087 vs. 0.074 ± 0.054, *p* = 0.019). However, this change was not observed in the poor Matsushima grade group (0.081 ± 0.056 vs. 0.067 ± 0.051, *p* > 0.05).

**Table 3:** The comparison of baseline characteristics between the good post-operative collateral circulation group and the poor group.

**Variables**	**Good group (*n* = 46)**	**Poor group (*n* = 39)**	***P*-Value**
Age	32.75 ± 1.24	33.52 ± 1.31	0.847
Female	20 (55.5%)	16 (44.5%)	0.820
Clinical history	26 (56.5%)	27 (69.2%)	0.228
PCA involvement	18 (32.6%)	6 (23.1%)	0.015
**Genotype of p.R4810K**			**0.298**
G/A	14 (30.4%)	8 (20.5%)	
G/G	32 (69.6%)	31 (79.5%)	
**Symptomatic presentation**			**0.040**
TIA	16 (34.8%)	14 (35.9%)	
Infarction	21 (45.7%)	8 (20.5%)	
Hemorrhage	6 (13.0%)	9 (23.1%)	
Headache, seizures	3 (6.5%)	8 (20.5%)	
**DSC-MRI on admission**			**0.043**
TTP delay (seconds)	4.79 ± 2.28	2.76 ± 1.68	
**EPC counts**			**0.019**
Before EDAS	0.074 ± 0.054	0.067 ± 0.051	
3 months post EDA	0.126 ± 0.087	0.081 ± 0.056	

## Discussion

In our study, we found that the heterozygous mutations of RNF213 p.R4810K were significantly correlated with spontaneous collateral circulation. In addition, we found that the CD34^bri^CD133^+^CD45^dim^KDR^+^ cell count in patient with post-EDAS was significantly higher as compared to the count before EDAS in the good Matsushima grade group. This implied that the mutations of RNF213 p.R4810K affect the establishment of spontaneous collateral circulation, while CD34^bri^CD133^+^CD45^dim^KDR^+^ cells are involved in the process of newly formed EDAS collaterals. To the best of our knowledge, this is the first report to explore the mechanism of these two types of collateral vessels in MMD.

Recently, several studies have indicated that in patients the homozygous c.14429G>A (p.R4810K) variant, rather than the heterozygous variant, was significantly associated with a young age at disease onset, cerebral infarction at presentation, and poor cognitive outcomes (22, 23, 34–36). However, we found that patients carrying heterozygous mutations of *RNF213* p.R4810K had a more severe pattern, such as infarction on presentation, more severe hemodynamic impairment, worse neurological status, and good post-operative Matsushima grade in our study (Table 4). One explanation could be that our cases were collected from a group with homogeneous age (30–35 years), while the homozygous p.R4810K variant of *RNF213* always predicts early onset of MMD in patients (23, 36). Patients with a young age at disease onset themselves have a more severe clinical course of MMD (37, 38). Another explanation could be that the patients with heterozygous mutations had a higher rate of PCA involvement (50%) in our study, which was similar to that of a previous Chinese study (48.7%) (39).

**Table 4:** The comparison of baseline characteristics between the G/A group and the G/G group.

**Variables**	**G/A group (*n* = 22)**	**G/G group (*n* = 63)**	***P*-Value**
Age	32.48 ± 1.16	33.67± 1.41	0.712
Female	8 (36.3%)	28 (44.5%)	0.509
History of risk factors	15 (68.1%)	38 (60.3%)	0.512
**Symptomatic presentation**			**0.341**
TIA	6	24	
Infarction	11	18	
Hemorrhage	3	12	
Headache, seizures	2	9	
PCA involvement	11	13	0.008
**Neurological status**			**0.431**
Good	19	58	
Poor	3	5	
**DSC-MRI on admission**			
TTP delay (seconds)	5.17 ± 2.61	2.42 ± 1.53	0.042
EPC counts before EDAS	0.078 ± 0.061	0.069 ± 0.053	0.087

*TIA, transient ischemic attack; PCA, posterior cerebral artery; DSC-MRI, dynamic susceptibility contrast-magnetic resonance imaging; TTP, time to peak; EPC, endothelial progenitor cell; EDAS, encephaloduroarteriosynangiosis*.

In fact, severe MMD with an unfavorable prognosis has been attributed to insufficient compensatory collateralization, which plays a pivotal role in the pathophysiology of cerebral ischemia. The collateral circulation, especially the leptomeningeal system, plays the most important role in the collateral supply of the ischemic cortex in the ACA and MCA territory in patients with MMD (40). Recently, we proposed a new collateral grading system to capture the entire scope of spontaneous collateral circulation, aiming to assess the intracranial blood supply (31). We found that in patients with good leptomeningeal collateral that there was a prolonged TTP as compared to normal but that in those with poor LM collateral the TTP was further prolonged. Further, the heterozygous mutations were significantly correlated with poor collateral circulation, which may be the cause of the severe symptoms and worse neurological status. These findings suggest that not only homozygous but heterozygous c.14429G>A (p.R4810K) may be useful as a specific biomarker for severe MMD with an unfavorable prognosis that requires timely intervention for revascularization surgery.

Indirect cerebral revascularization through EDAS was demonstrated to establish new collateral vessels through the formation of vessels from the external carotid artery (ECA) to the internal carotid artery (ICA) in patients with MMD (5–9). Although this phenomenon has been extensively described in the literature, the mechanism involved in the formation of collateral vessels is poorly understood. In our study, good Matsushima grade was occurred significantly more frequently in those with more severe ischemia, such as lower spontaneous collateral grade and longer TTP delay, suggesting that ischemia and hypoxia are the main driving forces of the newly formed EDAS collaterals. In our study, we also found that the CD34^bri^CD133^+^CD45^dim^KDR^+^ cell count in patient post-EDAS was significantly higher as compared to the count prior to EDAS in the good Matsushima grade group. However, this change was not observed in the poor Matsushima grade group. The results supported the theory that ischemia and hypoxia can also increase the EPC count in the peripheral blood of patients, leading to angiogenesis (41). Hence, we infer that neovascularization induced by EDAS might involve the recruitment, proliferation, and assembly of CD34^bri^CD133^+^CD45^dim^KDR^+^ cells and their ability to promote angiogenesis.

Spontaneous collateral circulation, or native collaterals, occurs through a process known as arteriogenesis, which is flow-dependent and hypoxia-independent. In arteriogenesis, pre-existing vessels with stenotic segments are exposed to increased shear stress due to a high-pressure gradient between two vascular territories (42). Arteriogenesis involves the recruitment and enlargement of pre-existing vessels, and sprouting and splitting are not parts of this process. A previous study showed that when a collateral develops into a vessel distal to a stenosis, higher flow and increased shear stress are associated with the enlargement of the collateral vessel (11). We found no significant difference in the CD34^bri^CD133^+^CD45^dim^KDR^+^ cell count among the three spontaneous collateral circulation groups, although the heterozygous c.14429G>A (p.R4810K) of RNF213 was significantly correlated with spontaneous collateral circulation. Knockdown of RNF213 in zebrafish leads to abnormal sprouting and irregular diameter of intracranial vessels, suggesting its possible contribution to vascular formation (21). These findings suggest that mutations of *RNF213* p.R4810K may inhibit spontaneous collateral circulation. However, the pathophysiological role of RNF213 and the mechanisms by which RNF213 polymorphisms lead to MMD have not been elucidated. Further research on its contribution to the pathogenesis of MMD is necessary.

The seminal findings of Asahara et al. describe the isolation and characterization of putative EPCs (14). EPCs play an essential role in the integrity and functionality of neo-vasculature under specific programmatic pathways that include angiogenesis and are especially relevant for tissue engineering strategies (43, 44). Since its initial discovery, the phenotypic definition of EPCs has undergone a gradual transformation to include a wide number of phenotypic cell surface markers (45, 46). The ongoing stratification and sub-stratification of cell surface markers have made inter-laboratory findings regarding tissue neo-vascularization difficult to compare at both the qualitative and quantitative levels. More recently, experts in the field of lymphatics and vascular biology offer a perspective article attempting to initiate consensus terminology for EPCs along with their phenotypic expression (16). However, numerous peer-reviewed studies that span decades have utilized different cell surface markers to demonstrate these EPCs to incorporate into neo-vasculature (18, 47–50). Within this context, we have utilized a population of cells defined as CD34^bri^CD133^+^CD45^dim^KDR^+^ (18, 19). Our study suggested that the CD34^bri^CD133^+^CD45^dim^KDR^+^ cells might play a significant role in the formation of collaterals post-surgery, which is a potential mechanism behind therapeutic collaterals in MMD.

This study had certain limitations that should be noted. First, it remains unknown whether CD34^bri^CD133^+^CD45^dim^KDR^+^ cells are myeloid angiogenic cells (MACs) as defined by Medina et al. (16), since we characterized them based on a different set of markers. In addition, *in vitro* or *in vivo* testing was not performed to assess whether the CD34^bri^CD133^+^CD45^dim^KDR^+^ cells exhibit endothelial properties and could indeed contribute to angiogenesis. The identification of these cells and their role in the formation of collateral circulation in MMD are worthy of further study.

Secondly, we only analyzed the relationship between RNF213 p.R4810K and the two types of collateral vessel formation. However, MMD is a complex disease with genetic heterogeneity and a classic pattern of inheritance, and therefore the single gene-focused study cannot sufficiently elucidate its genetic susceptibility. Therefore, future studies should include other mutations to comprehensively determine the genetic basis of this pathology. Thirdly, this study was based on a single-center hospital, so selection bias was unavoidable. Fourthly, we only enrolled patients who were aged 30–35 years, which may also have contributed to an unavoidable selection bias. Finally, the value of cerebral hemodynamics determined by perfusion MRI was relative and not absolute, and further technical refinements are required to enhance its diagnostic value.

## Conclusion

Our study implied that the mutations of RNF213 p.R4810K affect the establishment of spontaneous collateral circulation, while CD34^bri^CD133^+^CD45^dim^KDR^+^ cells are involved in the process of newly formed EDAS collaterals. Heterozygous c.14429G>A (p.R4810K) may be useful as a specific biomarker for severe MMD with an unfavorable prognosis that requires timely intervention for revascularization surgery.

## Data Availability Statement

The datasets presented in this article are not readily available due to ethical and privacy restrictions. Requests to access the datasets should be directed to the corresponding author.

## Ethics Statement

The studies involving human participants were reviewed and approved by the Research Ethics Board at the Fifth Medical Center of Chinese PLA General Hospital. The patients/participants provided their written informed consent to participate in this study.

## Author Contributions

X-YB: drafting the article. Y-NF: acquisition of data and analysis and interpretation of data. Q-NW: acquisition of data and statistical analysis. X-PW, R-MY, and Z-XZ: acquisition of data. QZ and D-SL: technical support. X-GY and LD: conception and design and critically revising the article. All authors contributed to the article and approved the submitted version.

## Funding

This study was supported by a grant from the National Natural Science Foundation of China (Grant No. 82171280) and the Hygiene and Health Development Scientific Research Fostering Plan of Haidian District Beijing (HP2021-04-80202).

## Conflict of Interest

The authors declare that the research was conducted in the absence of any commercial or financial relationships that could be construed as a potential conflict of interest.

## Publisher's Note

All claims expressed in this article are solely those of the authors and do not necessarily represent those of their affiliated organizations, or those of the publisher, the editors and the reviewers. Any product that may be evaluated in this article, or claim that may be made by its manufacturer, is not guaranteed or endorsed by the publisher.
